# Dissecting systems-wide data using mixture models: application to identify affected cellular processes

**DOI:** 10.1186/1471-2105-6-177

**Published:** 2005-07-14

**Authors:** J Peter Svensson, Renée X de Menezes, Ingela Turesson, Micheline Giphart-Gassler, Harry Vrieling

**Affiliations:** 1 Department of Toxicogenetics, Leiden University Medical Centre, P.O. Box 9503, 2300 RA Leiden, the Netherlands; 2 Department of Oncology, Radiology and Clinical Immunology, Academic Hospital, 751 85 Uppsala, Sweden; 3 Department of Medical Statistics, Leiden University Medical Centre, P.O. Box 9604, 2300 RA Leiden, the Netherlands

## Abstract

**Background:**

Functional analysis of data from genome-scale experiments, such as microarrays, requires an extensive selection of differentially expressed genes. Under many conditions, the proportion of differentially expressed genes is considerable, making the selection criteria a balance between the inclusion of false positives and the exclusion of false negatives.

**Results:**

We developed an analytical method to determine a *p*-value threshold from a microarray experiment that is dependent on the quality and design of the data set. To this aim, populations of *p*-values are modeled as mathematical functions in which the parameters to describe these functions are estimated in an unsupervised manner. The strength of the method is exemplified by its application to a published gene expression data set of sporadic and familial breast tumors with *BRCA1 *or *BRCA2 *mutations.

**Conclusion:**

We present an objective and unsupervised way to set thresholds adapted to the quality and design of the experiment. The resulting mathematical description of the data sets of genome-scale experiments enables a probabilistic approach in systems biology.

## Background

Functional analysis of microarray data, e.g. to reveal enrichment of promoter sequences, metabolic pathways or signalling cascades, requires an extensive selection of differentially expressed genes. Arbitrarily chosen thresholds for fold-changes and/or significance of change are commonly used to split the genes in subsets of alternative (differentially expressed) and null (non-differentially expressed) genes, with acceptable proportions of false positives and false negatives. Which proportion is acceptable depends on the research question. For example, to find markers of a treatment or signatures of a mutation, it is usually enough to fix the FDR-control level [1], and select the most significant alternative genes. However, most differentially expressed genes will not be selected. For other purposes, e.g. when comparing differences between treatments or mutations, there is need for more thorough comparisons. For each gene we would like to know the probability of it being alternative or null. Such an approach will let us select genes that are truly – or truly not – differentially expressed, with associated estimates of false positives.

The differential expression of genes is evaluated after a comparison of gene expression levels, yielding a list of *p*-values. Commonly, a threshold is then fixed for either the number of genes selected, or for their *p*-value, and those genes corresponding to the *k *smallest *p*-values are classified as alternative. However, because of the large number of *p*-values involved, even if all features are null some small *p*-values may be observed due to pure chance. Thus, it is important to consider an estimate for the proportion of alternative features while determining the threshold.

The problem of estimating the proportion of alternative and null features has been handled by several authors [2-5]. In many experimental settings the alternative features make up a considerable fraction. This is a consequence of genes being connected in networks; altered expression of one gene can affect expression levels of multiple targets. Begley and co-workers [6] showed that the methylating agent MMS induced changes in the transcription level of 33% of the genes in the *S. cerevisiae *genome and oxidizing agent t-BuOOH altered 38% of the genes. In a study of familial and sporadic human breast cancers by Hedenfalk and co-workers [7], Storey and Tibshirani [5] estimated 33% of the genes to be differentially expressed between *BRCA1 *and *BRCA2 *mutation positive tumors. However, even though over 1,000 genes were considered to be changed, only 160 genes could be selected with a pFDR of 5% as a consequence of the overlap between alternative and null genes. It is intuitive that using only 16% of the differentially expressed genes for functional analysis is suboptimal, and we will show that this is indeed the case. To detect subtle but coordinated changes in gene expressions, it is advantageous to examine the joint behavior of inter-connected sets of genes [8], which implies the need for a generous selection. Significant changes in cellular processes can be detected in cases where the alteration of individual gene expressions is not significant [9].

The threshold determination has so far been left to the researcher. In practice it is either guided by limitations in the number of features to be verified after being classified as alternative, or by the desired false positive proportion in the final list. However, with the increasing rate at which new experiments are being performed, and decreasing cost of verification experiments, there will be a growing need for unsupervised ways of determining thresholds yielding a list of features with acceptable false positive and false negative rates.

We present here an objective and unsupervised way to set thresholds that are adapted to the quality and design of the experiment. Description of the distribution of both null and alternative *p*-values as mathematical functions will give the researcher the possibility to select genes depending on the probability of being alternative or null.

## Result

### A case study

**Figure 1:**
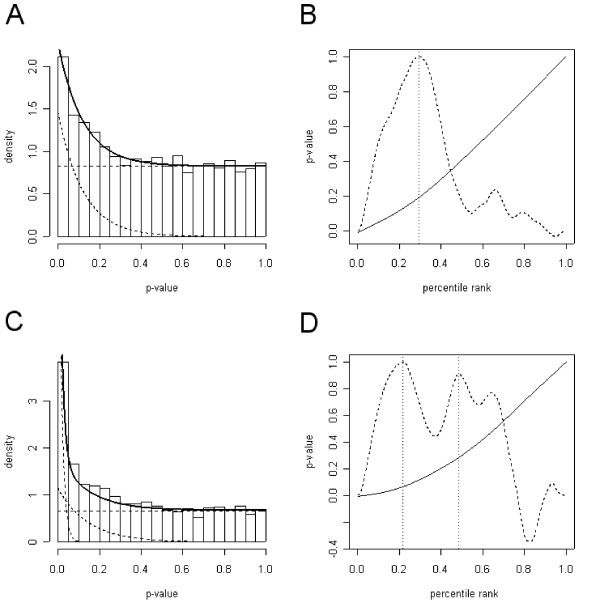
**Distributions of *p*-values from the Hedenfalk data set**. (A,C) Density histograms for the more than 3,000 genes comparing (A) *BRCA1*-mutated and sporadic tumors or (C) *BRCA1*- and *BRCA2*-mutated tumors. The dashed lines show the estimated distributions of the alternative (curves) and null (horizontal lines) genes, and solid lines show the sum the estimated distributions. (B,D) Scatterplots where the *p*-value of each gene is plotted against the percentile rank. A smooth function is fitted (solid line) and the local maxima of the curvature (dashed line) are used to split the null and alternative populations of genes for the comparisons (B) *BRCA1*-mutated and sporadic tumors or (D) *BRCA1*- and *BRCA2*-mutated tumors.

Hedenfalk et al [7] determined gene expression patterns in 21 tumors from breast cancer patients. Seven tumors were sporadic with unknown mutations, whereas the remainder were familial cancers, in which one of the known genes associated with breast cancer was mutated: *BRCA1 *(7 patients) or *BRCA2 *(8 patients). RNAs from the three different sources were hybridized to arrays containing 6,512 cDNA clones. After discarding low quality spots [5,7], more than 3,000 clones remained. The measurements from these genes were tested against the null hypothesis that there is no differential expression across two conditions [5]. The resulting *p*-values can be visualized in density histograms (figure 1A,C and supplementary material). The *p*-values appear to follow the expected distribution as being composed of a population of truly null (non-differentially expressed) genes with *p*-values uniformly distributed among [0, 1] and a population of truly alternative (differentially expressed) genes with *p*-values that tend to be close to zero. Similar shapes of distributions can be generated for all four tested comparisons, *BRCA1 *to sporadic, *BRCA2 *to sporadic, *BRCA1 *to *BRCA2 *and both *BRCA1 *and *BRCA2 *to sporadic.

### Decomposing distributions and setting thresholds

To further examine the gene expression responses, we describe the alternative and null distributions as mathematical functions. For this purpose, we plot the genes sorted by their *p*-values, *p*, against the percentile ranks (rank position/the total number of *p*-values), *y *(figure 1B) to reveal *F*^-1^, the inverse of the underlying cumulative density function. *F*^-1 ^is estimated non-parametrically. When comparing *BRCA1 *mutation-positive tumors against sporadic tumors,  is convex upward on the entire interval *y *= [0, 1]. The curvature  (dotted line in figure 1B) has a major peak (at *y*_0 _= 0.30 corresponding to *p*_0 _= 0.19) indicating that there is one dominating population of alternative genes. The expression for *f*(*p*_0_) gives us a rough  estimated to 0.77. Other methods specifically designed to estimate  yielded similar results; Storey and Tibshirani's method [5] led to  = 0.83 and the method developed by Schweder and Spjotvoll [4] resulted in  = 0.82. Under the assumption that the *p*-values of the alternative genes follow an exponential distribution, we calculate  to 8.3 (see methods section). With the parameters  and  we can describe the null and alternative density functions (dashed lines in figure 1A). To select differentially expressed genes, we use the critical point *p*_0 _as a *p*-value threshold. As a result, the null hypothesis was accepted for 2,083 genes with a *p *> 0.19. These genes all have at least a twice larger probability of being null than alternative. In general, since we have a mathematical description of *f*(*p*) any point can be chosen as threshold. For instance, we can calculate a point, *p*_1_, where genes have at least a two times larger probability of being alternative than null. Hundred-and-eighty genes have a *p*-value below this threshold of *p*_1 _= 0.04, and are selected as alternative. The proportion of false positives, as estimated by the ratio of integrals of *f*_0 _and *f *in the interval [0, *p*_1_], amounts to 20%. The pFDR estimated by Storey and Tibshirani [5] is 34%. Notably, in this comparison at a pFDR of 5%, being the proposed threshold by Storey and Tibshirani [5], no gene is selected as being differentially expressed.

Comparing expression data from *BRCA2 *and sporadic tumors shows similar patterns ( = 0.75 and  = 11.1) (supplementary figure 1). Here, using *p*_0 _as the *p*-value threshold 2,168 genes are selected as being null. Only 20% of the genes with *p *<*p*_0(*BRCA*1) _or *p *<*p*_0(*BRCA*2) _are in common in both comparisons, suggesting that *BRCA1 *and *BRCA2 *affect different target genes.

Information on the genes affected in both *BRCA1 *and *BRCA2 *mutation-positive tumors can be obtained by comparing the data from the familial tumors together against the sporadic. The curvature of , revealing the genes in common between *BRCA1 *and *BRCA2 *mutated tumors, has two local maxima (supplementary figure 1). Also when the data from *BRCA1 *is compared to that from *BRCA2 *mutated tumors (figure 1C–D), the curvature plot has several local maxima. These observations indicate that the original distributions might be constituted of *p*-values from two or more populations of truly alternative genes in addition to the truly null genes. Each of these groups is represented by a close to linear part in the plot of . The critical points of the curvature of  and the corresponding *p*-values let us estimate the parameters to describe the distributions of the groups.

### Simulation study

**Figure 2:**
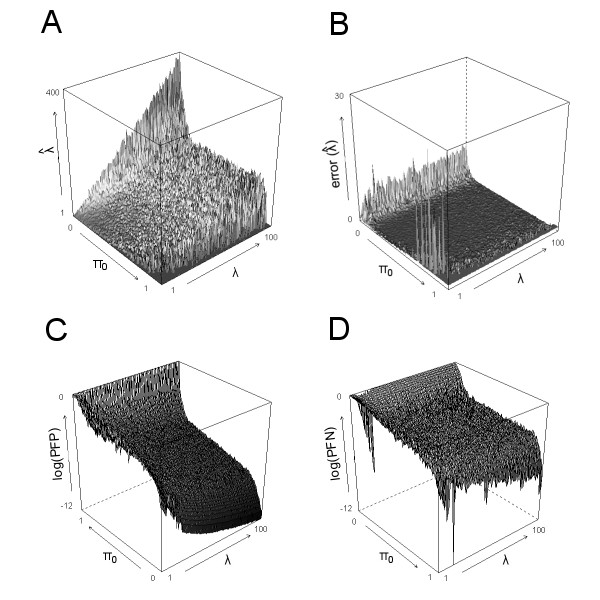
**Calculation of *p*_0 _and estimations of *λ *from a simulation study**. 10,000 *p*-values were simulated in a mixture of an alternative and a null distribution. As functions of *λ *∈ [1, 100] and *π*_0 _∈ [0, 1] are shown: (**A**) , (**B**) the relative error in , (**C**) the proportion of false positives (PFP) and (**D**) false negatives (PFN) when selecting the genes with *p *<*p*_0_. NB the inverse arrow of *π*_0 _in (**C**).

In order to verify how the method works in a case for which the result is known, a simulation study was performed. It was assumed for simplicity that each alternative *p*-value followed an exponential distribution with rate *λ*. For values of *λ *between 1 and 100, and varying proportions *π*_0 _of alternative *p*-values within [0, 1], a set of 10,000 independent *p*-values was generated. For each of these sets, an estimate  was computed for *π*_0 _using the method developed by Storey and Tibshirani [5]. Subsequently, an estimate  for *λ *was determined via a non-parametric approach (see methods section). A graph of the estimate  for all combinations of *λ *and *π*_0 _values, is shown in figure 2. The relative error in  was determined as  and the proportion of false positives and false negatives were calculated by  and  respectively.

The results show that  gives a reasonable estimate of *λ *for 0.20 <*π*_0 _< 0.95 and *λ *> 5 (figure 2). The proportion of false positives obtained for these values is 0.03 ± 0.01 (mean ± s.d.), and the proportion of false negatives is 0.04 ± 0.03. When few alternative features are present (*π*_0 _= 0.95), *λ *cannot be determined. Also when the alternative distribution is close to uniform (*λ *< 5),  fails to correctly estimate *λ*. For *π*_0 _= 0.20, *λ *is over-estimated and the method yields a conservative threshold, meaning that the proportion of false positives is low on the expense of a high proportion of false negatives. However, *π*_0 _being ≤ 0.20 is of relatively little practical interest, as typically by design, studies already contain a large fraction of unchanged genes.

**Figure 3:**
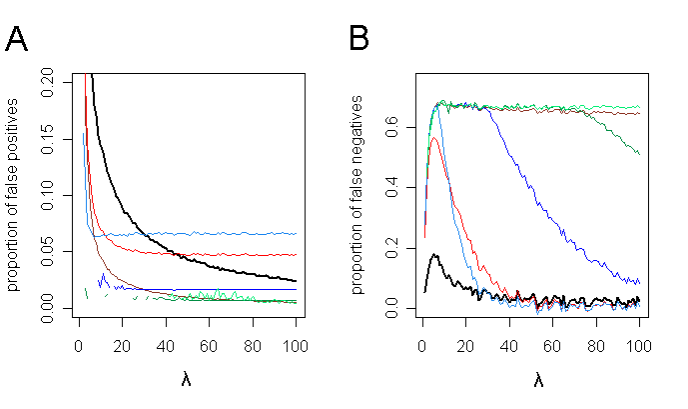
**Comparisons between *p*_0 _and other selection methods in a simulation study**. 10,000 *p*-values were simulated in a mixture of an alternative and a null distribution. The proportions of false positives (**A**) and false negatives (**B**) are depicted as functions of *λ *∈ [1, 100], *π*_0 _is set at 0.67. Selecting the genes with *p *<*p*_0 _(black) is compared to: selecting the genes with *p *< 0.001 (brown) or *p *< 0.1 (red); controlling the Benjamini-Hochberg FDR at *α *= 0.05 (dark blue) or *α *= 0.2 (light blue); the Benjamini-Yekutieli FDR at *α *= 0.2 (dark green) and the FWER at *α *= 0.2 (light green). The lines represent means of twenty simulations.

We also compared the proportions of false positives and false negatives between different methods for selecting genes, e.g. while controlling the family wise error-rate (FWER) or the FDR (figure 3). The proportion of false calls largely depends on the separation between alternative and null features (the value of *λ*) for thresholds based directly on the p-values. As to be expected, when correcting for multiple testing, i.e. controlling the FWER or FDR, the proportion of false positives remains constant.

Similar calculations can be made for complex populations with more than one population of alternative features.

### Functional analysis

To discern alternated cellular processes, we performed an analysis at gene set level [8,9]. To obtain a description of the function of the affected genes (*p *<*p*_0_) in the BRCA study, the genes on the arrays were associated with Gene Ontology (GO) terms [10]. The annotation of the genes in the original data files were updated to the Unigene 170 build (24 April 2004). Of the informative transcripts on the array, 62% could be attributed to gene sets that represent specific GO terms. Subsequently, the hypergeometric probability of the genes in each gene set being randomly distributed around the threshold, *p*_0_, was calculated.



where *x *is the number of affected genes in the gene set, *N *is the total number of affected genes, *n *is the total number of genes in the gene set and *m *is the total number of genes not in the gene set. In cases of several *p*_0_, we chose *p*_0 _corresponding to the major peak of the curvature plot, or if two major peaks are close to each other, the one associated with the largest *p*_0_.

The significant gene sets and the affected genes in the four comparisons are shown in supplementary tables 1–4.

When comparing *BRCA2 *and sporadic, only three gene sets were significantly (*p *< 0.01) altered, one consisting of genes involved in DNA repair. For *BRCA1*, 15 genes sets are significantly (*p *< 0.01) altered. The genes involve DNA binding, phosphorylation and cell cycle. Among the six enriched gene sets in the comparison between familial (*BRCA1 *and *BRCA2*) and sporadic tumors, the most significant enrichment is that of genes associated with the mitochondria. Almost as significant is the lack of electron transport genes among the selected genes.

We then tried to select genes using alternative methods. When controlling the FWER or the Benjamini-Yuketieli FDR, no genes were selected at the level *α *= 0.05 and when using the Benjamini-Hochberg FDR only the comparison between *BRCA1 *and *BRCA2 *yielded a non-zero list of genes. At the control-level *α *= 0.2, all four comparisons resulted in lists comprised of 2–450 genes, which is much less (familial versus sporadic) or comparable (BRCA1 versus BRCA2) to the number of genes selected as having *p *<*p*_0_. The significant genes sets after gene set analysis are partially overlapping with the results from the *p*_0 _threshold (supplementary tables 5–8).

**Figure 4:**
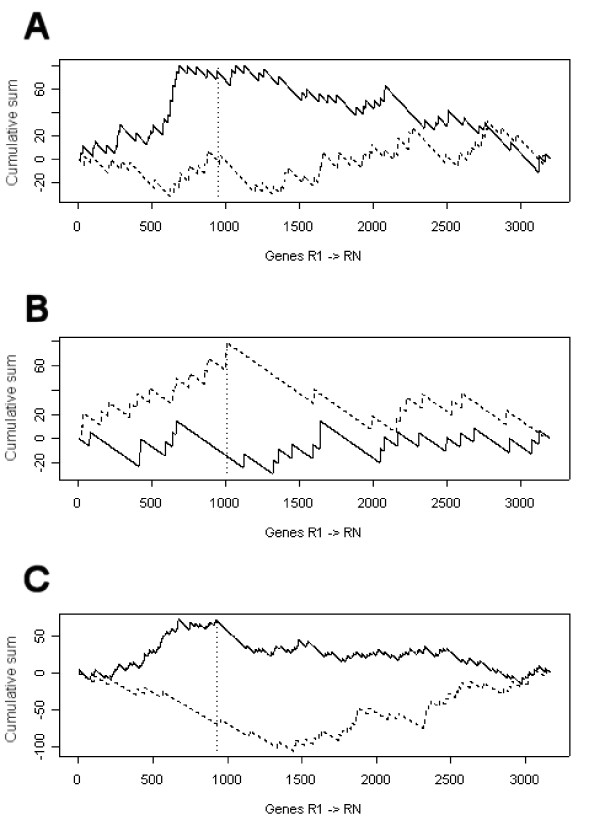
**Enrichment of gene sets in the BRCA-study**. Genes *R*_1_, ..., *R*_N _(*N *> 3,000) are ordered on their *p*-values and the cumulative sum is calculated to determine whether the members of a gene set are enriched. Starting with the top-ranking gene, the sum increases when a gene in the gene set is encountered and decreases otherwise. The gene sets shown here are (**A**) cell cycle (solid line – *BRCA1*-sporadic; dashed line – *BRCA2*-sporadic) and (**B**) DNA repair (solid line – *BRCA1*-sporadic; dashed line – *BRCA2*-sporadic). (**C**) For the comparison *BRCA1/2*-sporadic, the gene sets consisting of genes functional in the mitochondria (solid line) and electron transport (dashed line) are depicted. The vertical dotted lines correspond to the thresholds, *p*_0_, for *BRCA1*, *BRCA2 *and *BRCA1/2 *compared to sporadic.

For visualization of the distributions, we used a modified version of 'gene set enrichment analysis' [8], where the genes were sorted on the *p*-values to form a sequence. Genes belonging to the tested gene set are attributed a value of  and the remaining genes are given the value . The cumulative sum of the sequence is calculated and plotted for a few gene sets in figure 4. The more a gene set deviates from a uniform distribution, the greater absolute value of the sum. For these gene sets, the threshold at *p*_0 _coincides with the maximum of the cumulative sum.

## Discussion

High-throughput techniques such as gene expression profiling by microarrays allows rapid screening of large amounts of data simultaneously. However, the staggering amount of data produced causes new problems, such as how to determine a meaningful *p*-value threshold between differentially expressed and unchanged genes. Methods such as Benjamini & Hochberg's [1] or Benjamini & Yekutieli's [11] control false positives proportions, but yield no information about false negatives proportions. Our method can produce a full description of the probability density function for differentially expressed genes, which makes it possible to rationally choose a desired ratio between *π*_0 _and 1 - *π*_0 _for which the *p*-value threshold can be calculated.

The most important features of our proposed *p*-value threshold are that it is data dependent and unsupervised. It relies on an independent estimate of the unchanged genes proportion, . The method works best for *π*_0 _∈ (0.20,0.95) and *λ *> 5, situations in which the number of alternative features is non-negligible and has a minimum separation from the null features.

The value of *λ *will increase with the separation of the populations of alternative and null genes. The power of the statistical test will also be reflected on *λ*, as well as the quality of the data. On commercial platforms with multiple synthetic probes, *λ *tends to be higher than on microarrays spotted with PCR products (data not shown).

As the tolerable amount of false calls depends on the research question, relaxed selection criteria are needed for questions which require a balance between the inclusion of false positives and the exclusion of false negatives. Conventional ways of selecting genes, such as stringent control of the FWER or FDR, select few, if any, false positives whereas the proportion of false negatives is at its maximum even with good separation between alternative and null features (figure 3). Only when relaxing the thresholds are we able to bring the two rates into equilibrium. Ideally, with no overlapping populations between truly alternative and truly null genes and a single population of alternative genes, the threshold at *p*_0 _will select  of the total genes as differentially expressed. However, in practice there is always some overlap in *p*-values between truly alternative and truly null genes. Selection of genes with *p *<*p*_0 _will frequently include many genes which can be useful under some circumstances. For instance, although in the examples of the case study the FDRs are high at *p*_0_, almost all alternative genes are included, as can be estimated by . In the functional analysis, the plots of the cumulative sums show that setting the threshold at *p*_0 _is biologically valid. Up until these points there is still a contribution of genes in the relevant gene sets. A more stringent selection would neglect the evidence of many genes being marginally differentially expressed. By selecting genes through controlling the FDR, similar results can be obtained with relaxed thresholds. The analysis of biological function that we present on the BRCA data set was expected to reveal groups of genes related to the cellular processes that are affected by either mutant BRCA1 or mutant BRCA2. Indeed for certain predefined gene sets, e.g. genes involved in DNA repair, cell cycle progression or nucleic acid interactions, the proportion of genes with *p *<*p*_0 _is higher than can be expected from a random distribution. The observation that the threshold *p*_0 _coincides with the maximum of deviation from zero in figure 4 suggests that *p*_0 _is at the breakpoint where the enrichment of the gene set culminates. We noticed that there are more gene sets significantly affected by mutated *BRCA1 *than mutated *BRCA2*. Next to "protein phosphorylation", specifically gene sets involved in cell cycle regulation are enriched, including "cell cycle", and the somewhat less significantly induced gene sets "mitosis" and "spindle". As for mutated *BRCA2*, there is no indication that its impairment affects cell cycle progression; the genes in the gene set "mitosis" appear even to be depleted among the affected genes. The involvement of DNA repair is additionally endorsed by the less significantly induced gene sets "response to stress" and "induction of apoptosis by extracellular signals". From the literature we know that the BRCA network deals with lesions that block or interfere with DNA replication. BRCA1 has a role in DNA damage sensing but its precise function is not known (for review see [12]). BRCA2 is directly involved in DNA repair [13]. Interestingly, when *BRCA1 *and *BRCA2 *mutation positive tumors are compared together against sporadic tumors, mitochondrial genes are affected but not electron transport genes. Until recently, the main function of mitochondria was thought to be the provision of energy for the cell through the creation of an electrical potential across its membrane. However, mitochondria are also involved in one route of apoptosis and recently there has been evidence for dysfunctional mitochondria being associated with premature ageing [14]. These are functions that could possibly be connected to the BRCA network.

## Conclusion

The method presented here will allow researchers to set unsupervised thresholds to select alternative features from mixed populations of *p*-values. As the discipline of systems biology evolves, there will be a need to compare global measurements of different levels (RNA, proteins, metabolites, etc.). The evidence of features being alternative can be used as weights in a comparison. In this context, a mathematical description of significant features enables a probabilistic approach to identify affected pathways.

## Method

### Setting thresholds

The *p*-values are defined as independent continuous random variables *P*_1_, *P*_2_, ..., *P*_*n *_taking values in the interval [0, 1]. Let us represent by *f *and *F *the probability density and cumulative probability functions of the distribution of a generic *p*-value *P*, respectively. We require that *f *is twice differentiable.

We represent by *f*_0_(*p*) and *f*_1_(*p*) the *p*-value densities for a null feature and an alternative feature, respectively. For a generic feature, its *p*-value density can be written as the mixture

*f*(*p*) = *π*_0_*f*_0_(*p*) + (1 - *π*_0_) *f*_1_(*p*), ∀ *p *∈ [0, 1],     (1)

where *π*_0 _represents the proportion of null features out of the total under study. Note that *f*_0_(*p*) takes the value 1 for all *p *within [0, 1].

A common way of visualizing all *p*-values is to make a graph of the sorted *p*-values according to the features (see for example the solid line in figures 1B and 1D). If there are no alternative features, this line should roughly be a straight line with a 45 degrees angle. The presence of alternative features makes the line more convex, as seen in our examples. This line corresponds to *F*^-1^, where *F *is the cumulative probability function as defined above.

We represent by *p*_0 _the *p*-value threshold defining the largest *p*-value corresponding to features identified as alternative. We shall define *p*_0 _as the maximum of the second derivative of *F*^-1^. The second derivative correspond to the curvature of the original function. Using (1), we get that *p*_0 _satisfies



Note that there need not be a unique value of *p*_0 _satisfying (2). The derivation of the equation is given in the appendix.

For an intuitive understanding, consider the extreme case where the alternative features under study all have *p*-values equal to zero, whereas the null features have a uniform distribution over [0, 1]. Then *F *has probability mass at *p *= 0 equal to the proportion of alternative features (1 - *π*_0_), and in the interval (0, 1] is described by a straight line between (0, 1 - *π*_0_) and (1, 1). It is clear that the best threshold *p*_0 _is the one that selects all features with zero *p*-values. This corresponds to the turning point of *F*^-1 ^In practice, the alternative features will not all have zero *p*-values, but the turning point of *F*^-1^, where its second derivative is zero, remains the best threshold to yield a compromise between false positives and false negatives proportions.

### Application to exponential distributions

To give some insight about how the proposed threshold works, let us consider the case when the alternative *p*-values have an exponential distribution. This consists of one of the simplest functional forms for a monotonically decreasing density within [0, 1] with probability density function given by

*f*_1_(*p*) = *G*(*λ*)*e*^-*λp*^, 0 ≤ *p *≤ 1,     (3)

where , a constant guaranteeing that . As *λ *→ 0, *f*_1 _approaches the uniform distribution. Equation (2) yields



If we replace this into (3), we obtain



and, by replacing the latter into expression (1) for *f*, we get



Thus, the threshold is set at a point where the proportion of the alternative features is half the proportion of the null features. In other words, the genes with a *p*-value less than this threshold all have some evidence against the null hypothesis, whereas very few truly differentially expressed genes are excluded. For many purposes this definition gives a too wide selection, and it is more desirable to have a point where the proportion of alternative genes is higher than the proportion of null genes, e.g. at a point *p*_1 _where . At *p*_1_, the proportion of alternative genes is twice the proportion of null genes. However, with the functional description given above, any point depending on the ratio between the proportions of null and alternative genes can be found in an unsupervised manner.

**Figure 5:**
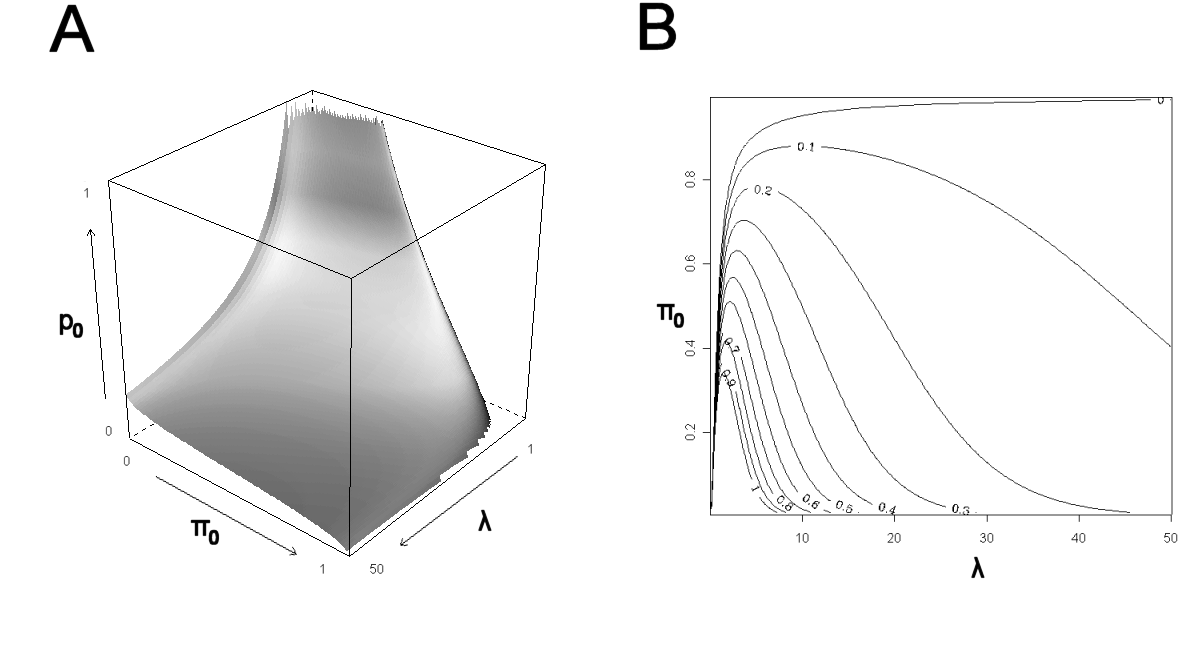
**The *p*-value threshold dependencies on *π*_0 _and *λ***. *p*-values come from a mixture of *π*_0 _null genes and a single population of 1 - *π*_0 _alternative genes following an exponential distribution  visualized in (A) a 3D plot and (B) a contour plot of *p*_0_.

The dependency of the threshold *p*_0 _on *λ *and *π*_0_, is visualized in figure 5. In this figure, it is clear that the suggested threshold *p*_0 _varies with *π*_0 _and *λ *in the desired way: as *λ *increases, the separation between the null and alternative *p*-values distributions increases and, therefore, *p*_0 _decreases. On the other hand, as the proportion of null genes *π*_0 _increases, naturally it becomes harder to identify the alternative genes, and again *p*_0 _decreases.

For practical reasons it is desirable to have an estimate of the proportion of false positives in the selection. Provided the described functions, we can integrate *f*_0 _and *f *in the interval [0, max(*p*)]. The ratio between the two will be an estimate of the proportion of false positives.

Extensions to more than one alternative component are straightforward and given in appendix. Application of the more flexible beta distribution yield comparable results (see appendix).

### Non-parametric approach

In practice, there is interest in making as few distributional assumptions as possible so as to yield a robust approach. We suggest estimating *F *non-parametrically. This can be done by plotting the sorted *p*-values of all genes against their percentile rank (as the scatterplots in figures 1B and 1D) and estimating *F*^-1 ^by fitting a smooth function – such as a cubic spline – to the data points. The smoothing parameters are chosen by cross-validation.

In non-parametric regression, the equivalent degrees of freedom for noise (*EDF*) is defined by *EDF *= *tr*{*I *- *A*(*α*)}, where *A*(*α*) is the hat matrix associated with spline smoothing with smoothing parameter *α*. The relation between *EDF*, the generalized cross validation (*GCV*) score and the residual sum of squares [15,16] can be written as



The optimal *EDF*, and thus smoothing parameter, to fit the smoothing spline is found by minimizing the residual sum of squares.

To get the curvature of the spline, , it is differentiated numerically twice. The critical points *p*_0_, of this second derivative can be identified.

The R code and additional information is available at .

## Authors' contributions

JPS designed and implemented the method and drafted most of the manuscript. RXM accounted for statistical expertise and substantial parts of the draft. MGG, IT and HV supervised the study and participated in manuscript writing. All authors read and approved the final manuscript.

## Supplementary Material

Additional File 1 **Supplementary tables 1–4**. Gene sets enriched in the comparisons among familial (*BRCA1 *and *BRCA2 *mutation positive) and sporadic tumors selecting the genes with *p *<*p*_0_. Click here for file

Additional File 2 **Supplementary tables 5–8**. Gene sets enriched in the comparisons among familial (*BRCA1 *and *BRCA2 *mutation positive) and sporadic tumors, controling the Benjamini-Hochberg FDR at *α *= 0.2. Click here for file

Additional File 3 **Supplementary figure 1**. Histograms and scatterplots of *p*-values from comparisons between *BRCA2 *mutation positive and sporadic tumors, and between *BRCA1 *and *BRCA2 *mutation positive and sporadic tumors. Click here for file

Additional File 4 **Appendix**. Calculation of derivatives and the application to exponential and Beta distributions. Click here for file

Additional File 5 **The pseudo-code and R code**. Methods to calculate *p*_0 _and estimate *f*(*p*) given a vector of *p*-values. Click here for file
