# Supplementary figures and images for: Dissecting systems-wide data using mixture models: application to identify affected cellular processes
Source: BMC Bioinformatics. 2005 Jul 14;6:177. doi: 10.1186/1471-2105-6-177 (PMC1189081; doi:10.1186/1471-2105-6-177)

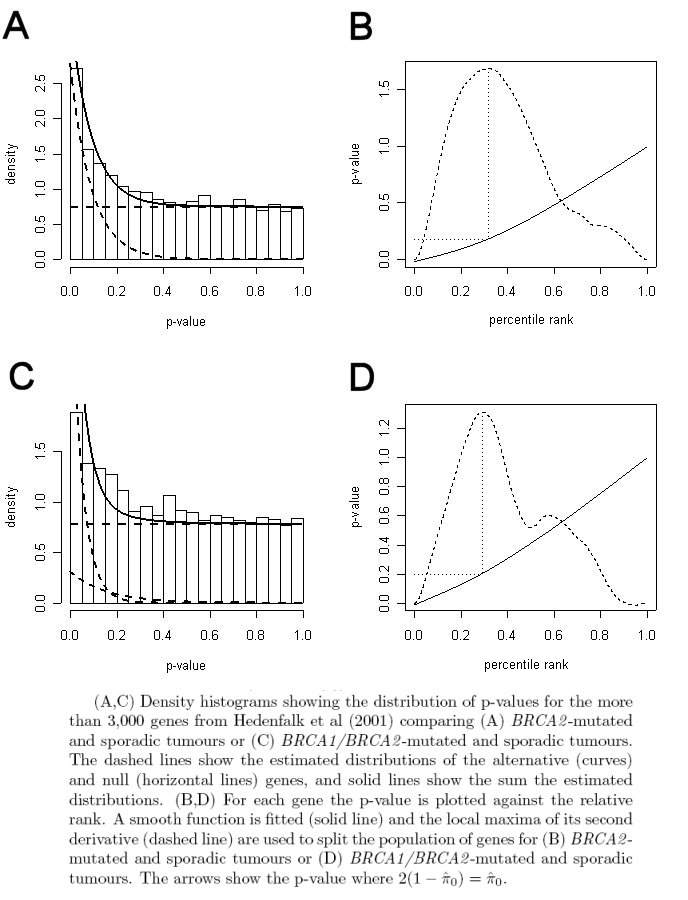

Supplement: Additional File 3 — Supplementary figure 1. Histograms and scatterplots of p-values from comparisons between BRCA2 mutation positive and sporadic tumors, and between BRCA1 and BRCA2 mutation positive and sporadic tumors. [file 1471-2105-6-177-S3.bmp]
