# Supplementary material for: Dissecting systems-wide data using mixture models: application to identify affected cellular processes
Source: BMC Bioinformatics. 2005 Jul 14;6:177. doi: 10.1186/1471-2105-6-177 (PMC1189081; doi:10.1186/1471-2105-6-177)
Supplement: Additional File 4 — Appendix. Calculation of derivatives and the application to exponential and Beta distributions. [file 1471-2105-6-177-S4.pdf]

Supplementary material for "Dissecting systems-wide data using mixture models: application to differentially expressed genes in the BRCA network"

Peter Svensson[1,2], Renée X. de Menezes[3], Ingela Turesson[2], Micheline Giphart-Gassler[1], Harry Vrieling*[1]

[1]Department of Toxicogenetics, Leiden University Medical Centre, P.O. Box 9503, 2300 RA Leiden, the Netherlands
[2]Department of Oncology, Radiology and Clinical Immunology, Academic Hospital, 751 85 Uppsala, Sweden
[3]Department of Medical Statistics, Leiden University Medical Centre, P.O. Box 9604, 2300 RA Leiden, the Netherlands

Email: Peter Svensson - p.svensson@lumc.nl; Renée X. de Menezes - r.x.menezes@lumc.nl; Ingela Turesson - ingela.turesson@onkologi.uu.se; Micheline Giphart-Gassler - m.giphart-gassler@lumc.nl; Harry Vrieling*- h.vrieling@lumc.nl;

*Corresponding author

1 Calculation of derivatives

The probability density function, $f_p(p)$is used as in

$$P\{p \in [0,\delta]\} = \int_{[0,\delta]} f_p(p)\mathrm{d}p \tag{1}$$

which is related to the cumulative density function (probability function) as

$$y = F_p(p) = \int_0^p f_p(u)\,\mathrm{d}u \tag{2}$$

The mirror of the function is

$$p = F^{-1}(y) = g(y) \tag{3}$$

where $F^{-1}(p)$ is the inverse of $F(p)$. We are interested in the maximum of the second derivative of $g(y)$, i.e. where the third derivative $g'''(y)$ is zero[1].

$$g'''(y) = \frac{3f'(p)^2 - f(p)f''(p)}{f(p)^5}\Big|_{p=F^{-1}(y)} \tag{4}$$

Under the condition $g'''(y_0) = 0$, we get

$$f(p_0)f''(p_0) = 3f'(p_0)^2 \tag{5}$$

[1]

$$g'(y) = \frac{\mathrm{d}}{\mathrm{d}y}g(y) = \frac{\mathrm{d}}{\mathrm{d}y}F^{-1}(y) = \frac{\mathrm{d}p}{\mathrm{d}y} = \frac{1}{f(p)}\Big|_{p=F^{-1}(y)}$$

$$\begin{aligned} g''(y) &= \frac{\mathrm{d}}{\mathrm{d}y}g'(y) = \frac{\mathrm{d}}{\mathrm{d}y}\frac{\mathrm{d}p}{\mathrm{d}p}\frac{1}{f(p)} = \frac{\mathrm{d}p}{\mathrm{d}y}\frac{\mathrm{d}}{\mathrm{d}p}\frac{1}{f(p)} = \frac{1}{f(p)}f'(p)\frac{-1}{f(p)^2} = \\ &= -\frac{f'(p)}{f(p)^3}\Big|_{p=F^{-1}(y)} \end{aligned}$$

2 Exponential distributions

The density function arising from a mixture of distributions in general can be written $f(p) = \sum_k \pi_k f_k(p)$ for $f(p)$ consisting of k distributions, each affecting a proportion of π_k genes. $f(p) = \frac{\lambda}{1-e^{-\lambda}} e^{-\lambda p}$ where $\lambda \geq 0$ and $0 \leq p \leq 1$. The distribution of the p-values of the null features,$f_0(p)$, are uniformly distributed over $p = [0, 1]$.

As $f(p)$ is a sum of independent functions, the derivatives will also be sums of functions.

$$f'(p) = \sum_k \pi_k f_k'(p), \quad f''(p) = \sum_k \pi_k f_k''(p) \tag{6}$$

At the point p_0 where $g'''(y_0) = 0|_{y_0=F^{-1}(p_0)}$. From equation (5) follows[2]

$$\pi_0 f''(p_0) = 2(1-\pi_0)f'(p_0)^2 - \sum_{i<j\in[1,k]} \pi_i \pi_j \big(f_i(p_0)f_j(p_0)\big)', \tag{7}$$

at the point p_0 where $g'''(y_0) = 0|_{y_0=F^{-1}(p_0)}$ If $\lambda_i \ll \lambda_j$ the second term becomes negligible. The roots of equation (7) will provide equations to determine λ_i and π_i for $i \in [1, k]$. Depending on $f(p)$ there may be several roots to equation (5).

In the special case where the p-values come from a mixture of null features with a uniform distribution, $f_0(p)$, and a single population of alternative features following the exponential distribution $f_1(p)$, then $f(p) = \pi_0 + (1-\pi_0)f_1(p)$. Equation (5) leads to

$$f_1(p_0) = \frac{\pi_0}{2(1-\pi_0)} \tag{8}$$

With $f(p_0) = \frac{3}{2}\pi_0$, we can read off an estimate of π_0 from the density function, as two thirds of $f(p_0)$. There are several other ways described to estimate π_0 and each methods will give slightly different results. We can estimate λ and fully describe the distribution, $f_1(p)$, making it possible to select any point as a threshold.

$$\begin{aligned}
g'''(y) &= \frac{\mathrm{d}}{\mathrm{d}y} g''(y) = -\frac{\mathrm{d}p}{\mathrm{d}y}\frac{\mathrm{d}}{\mathrm{d}p}\frac{f'(p)}{f(p)^3} = \\
&= -\frac{1}{f(p)} \frac{f(p)^3 f''(p) - 3f'(p)f(p)^2 f'(p)}{f(p)^6}\Big|_{p=F^{-1}(y)} = \\
&= \frac{3f'(p)^2 f(p)^2 - f(p)^3 f''(p)}{f(p)^7}\Big|_{p=F^{-1}(y)} = \\
&= \frac{3f'(p)^2 - f(p)f''(p)}{f(p)^5}\Big|_{p=F^{-1}(y)}
\end{aligned}$$

[2]

$$\begin{aligned}
\pi_0 &= \frac{3\Big(\sum_k \pi_k f_k'(p_0)\Big)^2 - \sum_{k\neq 0} \pi_k f_k(p_0) \sum_k \pi_k f_k''(p_0)}{\sum_k \pi_k f_k''(p_0)} = \\
&= \frac{2(\gamma_1\lambda_1 e^{-\lambda_1 p_0} + \gamma_2\lambda_2 e^{-\lambda_2 p_0} + \cdots + \gamma_k\lambda_k e^{-\lambda_k p_0})^2 \gamma_2 \lambda_2^2 e^{-\lambda_2 p_0}}{\gamma_1\lambda_1^2 e^{-\lambda_1 p_0} + \gamma_2\lambda_2^2 e^{-\lambda_2 p_0} + \cdots + \gamma_k\lambda_k^2 e^{-\lambda_k p_0}} - \\
&\quad - \frac{(\gamma_1\lambda_1 + \gamma_2\lambda_2)e^{-(\lambda_1+\lambda_2)p_0} - \cdots - (\gamma_1\lambda_1 + \gamma_k\lambda_k)e^{-(\lambda_1+\lambda_k)p_0} - \cdots}{\gamma_1\lambda_1^2 e^{-\lambda_1 p_0} + \gamma_2\lambda_2^2 e^{-\lambda_2 p_0} + \cdots + \gamma_k\lambda_k^2 e^{-\lambda_k p_0}} - \\
&\quad - \frac{(\gamma_{k-1}\lambda_{k-1} + \gamma_k\lambda_k)e^{-(\lambda_{k-1}+\lambda_k)p_0}}{\gamma_1\lambda_1^2 e^{-\lambda_1 p_0} + \gamma_2\lambda_2^2 e^{-\lambda_2 p_0} + \cdots + \gamma_k\lambda_k^2 e^{-\lambda_k p_0}} = \\
&= \frac{2\Big(\sum_k \pi_k f_k'(p_0)\Big)^2 - \sum_{i<j\in[1,k]} (\gamma_i\lambda_i + \gamma_j\lambda_j)^2 f_i(p_0) f_j(p_0)}{\sum_k \pi_k f_k''(p)} = \\
&= \frac{2(1-\pi_0)f'(p_0)^2 - \sum_{i<j\in[1,k]} \pi_i\pi_j \big(f_i(p_0)f_j(p_0)\big)'}{f''(p_0)}
\end{aligned}$$

The height of the maximum of the second derivative is increasing with λ on a reasonable interval. For a single exponential distribution of alternative genes,

$$g''(y_0)|_{y_0=F(p_0)} = -\frac{e^{2\lambda p_0}}{27\lambda(1-e^{\lambda})^2(1-\pi_0)^2}. \tag{9}$$

This means that $g''(y_0)$ is an indicator for the overlap between the populations of genes.

3 Beta distributions

The Beta distribution is defined on the closed interval $p = [0, 1]$, and described by two parameters, α and β.

$$f(p) = \frac{(1-p)^{\beta-1}p^{\alpha-1}}{B(\alpha,\beta)} = \frac{e^{(\beta-1)\log(1-p)+(\alpha-1)\log(p)}}{B(\alpha,\beta)} = \frac{e^{h(p)}}{B(\alpha,\beta)} \tag{10}$$

$$B(\alpha,\beta) = \frac{\Gamma(\alpha)\Gamma(\beta)}{\Gamma(\alpha+\beta)} \tag{11}$$

$$f'(p) = \frac{e^{h(p)}}{B(\alpha,\beta)}h'(p) \tag{12}$$

$$f''(p) = \frac{e^{h(p)}}{B(\alpha,\beta)}(h''(p)+h'(p)^2) \tag{13}$$

with

$$h(p) = (\beta-1)\log(1-p)+(\alpha-1)\log(p) \tag{14}$$

$$h'(p) = -\frac{\beta-1}{1-p}+\frac{\alpha-1}{p} \tag{15}$$

$$h''(p) = -\frac{\beta-1}{(1-p)^2}-\frac{\alpha-1}{p^2} \tag{16}$$

If we assume $f(p)$ to consist of a beta distribution of changed genes (f_1) and a uniform distribution of unchanged genes, π_0, equation (5) leads to

$$\pi_0\Big(h_1'(p_0)^2+h''(p_0)\Big) = (1-\pi_0)f_1(p_0)\Big(2h_1'(p_0)^2-h_1''(p_0)\Big) \tag{17}$$

If we restrict ourselves to explaining the distributions when $\alpha = 1$, the expression can be further simplified to

$$f_1(p_0) = \frac{\pi_0(\beta-2)}{(1-\pi_0)(2\beta-1)}, \quad \beta \neq \frac{1}{2} \tag{18}$$

For large values of β, $\frac{2\beta-1}{\beta-2} \approx 2$, and we end up with the same expression as for the exponential distribution.
